# Flexibility in Food Extraction Techniques in Urban Free-Ranging Bonnet Macaques, *Macaca radiata*

**DOI:** 10.1371/journal.pone.0085497

**Published:** 2013-12-20

**Authors:** Madhur Mangalam, Mewa Singh

**Affiliations:** 1 Biopsychology Laboratory, University of Mysore, Mysore, India; 2 Evolutionary & Organismal Biology Unit, Jawaharlal Nehru Centre for Advanced Scientific Research, Bangalore, India; CNR, Italy

## Abstract

Non-human primate populations, other than responding appropriately to naturally occurring challenges, also need to cope with anthropogenic factors such as environmental pollution, resource depletion, and habitat destruction. Populations and individuals are likely to show considerable variations in food extraction abilities, with some populations and individuals more efficient than others at exploiting a set of resources. In this study, we examined among urban free-ranging bonnet macaques, *Macaca radiata* (a) local differences in food extraction abilities, (b) between-individual variation and within-individual consistency in problem-solving success and the underlying problem-solving characteristics, and (c) behavioral patterns associated with higher efficiency in food extraction. When presented with novel food extraction tasks, the urban macaques having more frequent exposure to novel physical objects in their surroundings, extracted food material from PET bottles and also solved another food extraction task (i.e., extracting an orange from a wire mesh box), more often than those living under more natural conditions. Adults solved the tasks more frequently than juveniles, and females more frequently than males. Both solution-technique and problem-solving characteristics varied across individuals but remained consistent within each individual across the successive presentations of PET bottles. The macaques that solved the tasks showed lesser within-individual variation in their food extraction behavior as compared to those that failed to solve the tasks. A few macaques appropriately modified their problem-solving behavior in accordance with the task requirements and solved the modified versions of the tasks without trial-and-error learning. These observations are ecologically relevant – they demonstrate considerable local differences in food extraction abilities, between-individual variation and within-individual consistency in food extraction techniques among free-ranging bonnet macaques, possibly affecting the species’ local adaptability and resilience to environmental changes.

## Introduction

Animals respond to novel and unpredictable challenges in their physical and social environments by showing new or modified behavioral patterns, a phenomenon referred to as behavioral flexibility. Behavioral flexibility lies towards an extreme of a continuum of individuals’ plastic responses which range from developmental plasticity in physiology and anatomy to genetic changes accumulating over generations [1-3]. Variation in behavioral flexibility among populations and individuals results in phenotypic diversity, i.e., populations and individuals respond differently to different environmental conditions. Consequently, populations and individuals adapt to their surroundings to varying degrees, some better than others, to respond appropriately to a set of conditions [4-6]. Existing populations of non-human primates, other than responding appropriately to naturally occurring challenges, also need to cope with anthropogenic factors, such as environmental pollution, resource depletion, and habitat destruction [7-9]. Within such populations, adaptive variation in behavioral flexibility, or the expression of novel behavioral phenotypes, may have significant ecological and evolutionary consequences [10,11]. For example, ecological constraints on behavioral plasticity, through means of natural selection, may influence individuals’ responses to environmental heterogeneity, i.e., behavioral flexibility may increase or decrease, depending upon the adaptability of behavioral traits across a range of environmental conditions; overall, behavioral traits may vary between individuals but remain consistent within each individual [12-14].

Several animal species inhabit ecologically distinct habitats, wherein different environmental challenges need different strategies/techniques as selection favors only those that prove to be optimal or successful [4-6,15]. In the context of foraging, when conservative foraging techniques prove to be sub-optimal or unsuccessful, individuals may exploit the otherwise inaccessible food resources by developing novel extraction techniques, or refining the extant ones [16,17]. Extractive foraging abilities are ecologically more relevant for populations that are generalists or opportunists, or those that are exposed to altered or impoverished environments (and generally show a high degree of behavioral ﬂexibility in the context of foraging). Such populations and individuals within such populations are likely to show considerable intraspecific variations in food extraction abilities, some populations and individuals showing a greater range of behavioral flexibility than the others. Among mammalian species, non-human primates show a relatively high degree of behavioral flexibility [17-19], typically in the context of extractive foraging [16,20,21]. In particular, several studies examined variations in food extraction techniques (or the extent to which the animals exploit embedded food resources) among non-human primates, for example, variation in stone tool use by wild bearded capuchin monkeys [22], and wild chimpanzees [23,24], both of which involve social learning. However, to our knowledge, no existing study examines flexibility in food extraction techniques at a level which would provide information in the context of environmental changes related to human presence.

Investigating behavioral flexibility in non-human primates requires a species which inhabits a variety of habitats so that one can compare behavior(s) among populations of the same species (and among individuals within populations). One non-human primate species that inhabits a variety of habitats is the bonnet macaque, *Macaca radiata*. The bonnet macaque is endemic to Southern India. Apart from forests where the species feeds primarily on arboreal food resources, populations of bonnet macaques inhabit agricultural lands, roadsides and temples where they feed on naturally occurring food resources as well as on food items that humans offer them, or they obtain from leftovers in garbage heaps, or procure by raiding crops, houses, and shops [25]. One can study flexibility in food extraction techniques in bonnet macaques through naturalistic observations. However, naturalistic observations allow only a limited control over the conditions that might affect the behavior of individuals. Standardized experiments allow an appropriate control of extraneous variables, across both populations and individuals (under a set of conditions). Therefore, in this study we used the latter approach, wherein we presented urban free-ranging bonnet macaques with embedded food resources and observed their corresponding food extraction behavior, to examine (a) local differences in food extraction abilities, (b) between-individual variation and within-individual consistency in the problem-solving success and underlying problem-solving characteristics, and (c) behavioral patterns associated with higher efficiency in food extraction. Presuming that frequent exposure to novel, embedded food resources can stimulate the development of specialized food extraction abilities, we expected the macaques with more frequent exposure to novel physical objects in their surroundings to be more likely to solve novel food extraction tasks than those living under more natural conditions. These differences would affect the species’ local adaptability and resilience to environmental changes. A correlated set of individual behavioral traits, consistent over time and across situations, confers differential fitness consequences under divergent environmental conditions [26-28]; thus, we expected the macaques to show considerable between-individual variation and within-individual consistency in problem-solving success and the problem-solving characteristics. Moreover, because any learned behavior is generalizable to varied contexts [29], we expected the macaques to repeatedly use certain task features and modify their problem-solving behavior according to changes in those task features, revealing their knowledge of the task.

## Methods

### Ethics Statement

We observed the macaques from a distance as they followed their regular behavioral routine and as they solved the food extraction tasks which we presented them. Because we conducted our research on individuals which (a) did not belong to an endangered or a protected species, and (b) inhabited an unprotected land with an unrestricted public access, our research work did not require any legal permission. Nevertheless, we obtained an approval from the Institutional Animal Ethics Committee (IAEC) at the University of Mysore.

### Subjects and Study Sites

The subjects were 32 bonnet macaques: (a) 18 macaques (3 adult males, 4 juvenile males, 7 adult females, and 4 juvenile females) of a roadside group; and (b) 14 macaques (2 adult males, 4 juvenile males, 4 adult females, and 4 juvenile females) of a temple group. The two groups varied in their feeding ecology and niche structure. The macaques of the roadside group typically fed on naturally occurring resources, such as fruits, vegetables, and insects as well as on the food material obtained by raiding the surrounding cropland, whereas the macaques of the temple group fed on naturally occurring resources as well as on anthropogenic food items, such as biscuits, instant snacks, and soft drinks obtained as offerings or snatched from devotees and tourists. Consistent with the differences in feeding ecology, while we never observed the macaques of the roadside group handling an artificial object, we frequently observed the macaques of the temple group handling a wide variety of non-natural objects, mostly associated with food material (Mangalam and Singh, unpublished data). The two groups of macaques live ca. 3.7 km apart in Mysore, India (GPS coordinates: roadside group – 12°16'32"N 76°40'13"E, temple group – 12°14'41"N 76°40'55"E); the distance between the two groups was enough to prevent any transfer of individuals between them.

### Experimental Procedure

In order to examine local differences in food extraction abilities, we presented the macaques of the roadside and the temple group with two distinct food extraction tasks (time between the presentations of the two tasks for any individual, ca. 24 h): (a) task-1, a 400 ml unsealed PET bottle containing ca. 50 ml of sweet milk (Figure S1); and (b) task-2, a wire mesh box (dimensions: 7.5 cm X 7.5 cm X 17.5 cm) containing an orange (Figure S2). Presumably, task-1 was a novel task only for the macaques of the roadside group, whereas task-2 was a novel task for the macaques of both groups (we never observed the macaques of the roadside group handling an object even remotely resembling a PET bottle or a wire mesh box; however, we frequently observed the macaques of the temple group handling a PET bottle) (Mangalam and Singh, personal observation). We placed a task apparatus (task-1 /task-2) in the vicinity (ca. 1 m) of the focal macaque when there was no conspecific within at least 3m from itself. We video recorded the food extraction behavior of the macaque for the time during which the individual was in physical contact with the task apparatus. We scored the recorded video, wherein we documented whether the macaque (a) explored the task, i.e., physically inspected the apparatus; (b) attempted the task, i.e., forcibly tempered the task apparatus; or (c) solved the task, i.e., extracted food material from the task apparatus by means of any technique. Also, we documented work-time, i.e., the time spent handling the task apparatus measured as the time interval between the first and the last physical contact with the task apparatus.

In order to examine between-individual variation and within-individual consistency in food extraction abilities among the macaques of the temple group, we presented them six times successively with task-1 (time interval between any two successive presentations for any individual, mean ± se = 35.90 ± 23.61 h). Because only a few macaques of the roadside group could solve task-1/task-2 previously, they were very unlikely to show any detectable variation in their food extraction abilities; we thus excluded them from the second part of the study. We placed a task apparatus (task-1) in the vicinity (ca. 1 m) of the focal macaque when there was no conspecific within at least 3m from itself. We video recorded the food extraction behavior of the macaque for the time during which the individual was in physical contact with the task apparatus. We scored the recorded video, wherein we documented the solution technique, i.e., the technique used to successfully extract the food material from the PET bottle; and the following problem-solving characteristics: (a) Work-time (as defined above); (b) Number of bouts, i.e., the number of time stretches spent on different solving-techniques (i.e., manipulating bottle cap, neck, shoulder, body, or base) separated by periods of inactivity or exploration; (c) Duration of bouts; (d) Proportion of work-time spent on major solving-technique (we defined the major solving-technique as the solving-technique on which the macaque spent a greater proportion of work-time solving the task as compared to the other possible solving-techniques); (e) Proportion of work-time spent on exploration. Also, we determined the following individual characteristics of the macaque: (a) Age-class; (b) Sex; (c) Body mass; we allowed the macaque to climb a 1.5 m pole erected on an electronic weighing scale, and reach a baited semi-spherical vessel attached to the upper end of the pole; at the instant when the macaque reached for food material, we measured the body mass of the macaque to the nearest 10 g from the digital display of the weighing scale; (d) Dominance status (on interval scale, i.e., starting from an arbitrary defined zero point); we recorded macaques’ dyadic agonistic interactions with the conspecifics and used those recordings along with those of the conspecifics as described by Singh et al. [30] to determine the dominance score of the macaque on an interval scale; (e) Sociality; we recorded all activities of the macaque from 0801 to 1700 h using focal animal sampling method [31]; from the observations, we determined sociality as the proportion of daily time spent on aggressive and socio-positive behavior.

In order to examine behavioral patterns associated with higher efficiency in food extraction (i.e., shorter latency in food extraction), we presented the two temple group macaques AM1 and AF4 with manipulated versions of task-1: (a) task-1a, an unsealed PET bottle without cap-seal (Figure S3A); (b) Task-1b, an unsealed PET bottle with a relatively small cap-seal as compared to normal, such that there was a significant gap between cap and cap-seal (Figure S3B); (c) Task-1c, a PET bottle with an immovable cap and cap-seal (Figure S3C); (d) Task-1d, a PET bottle with a non-functional cap and cap-seal, such that even rotating the cap would not loosen the cap (Figure S3D). Earlier, AM1 and AF4 solved task-1 by opening bottle cap; thus we could examine whether they also possess the ability to modify their food extraction behavior to solve synonymous tasks without trial-and-error. Also, AM1 did not remove cap-seal earlier, as did AF4; we thus did not present AM1 with task-1a and task-1d – the tasks which would reveal an individual’s knowledge of cap-seal. We presented AM1 with task-1c and task-1d, and AF4 with task-1a, task-1b, task-1c and task-1d, each task for three times in a random sequence. Also, we presented the macaques of the temple group (including AM1 and AF4) that solved task-1 earlier with task-3, a 750 ml polycarbonate bottle without a cap-seal and with its cap positioned obliquely to the location of PET bottle cap (a task which was functionally similar to task-1 but would force the macaques to open bottle cap as it was nearly impossible for the macaques to puncture this polycarbonate bottle; Figure S4), and as before, observed the corresponding extraction behavior.

### Statistical Analysis

We did all statistical analysis using two-tailed tests on SPSS 20. We considered the outcomes of the tests statistically significant only when the value of alpha was lower than 0.05. We describe the details of the tests wherever we use them.

## Results

### Local Differences in Food Extraction Abilities

**Figure 1:**
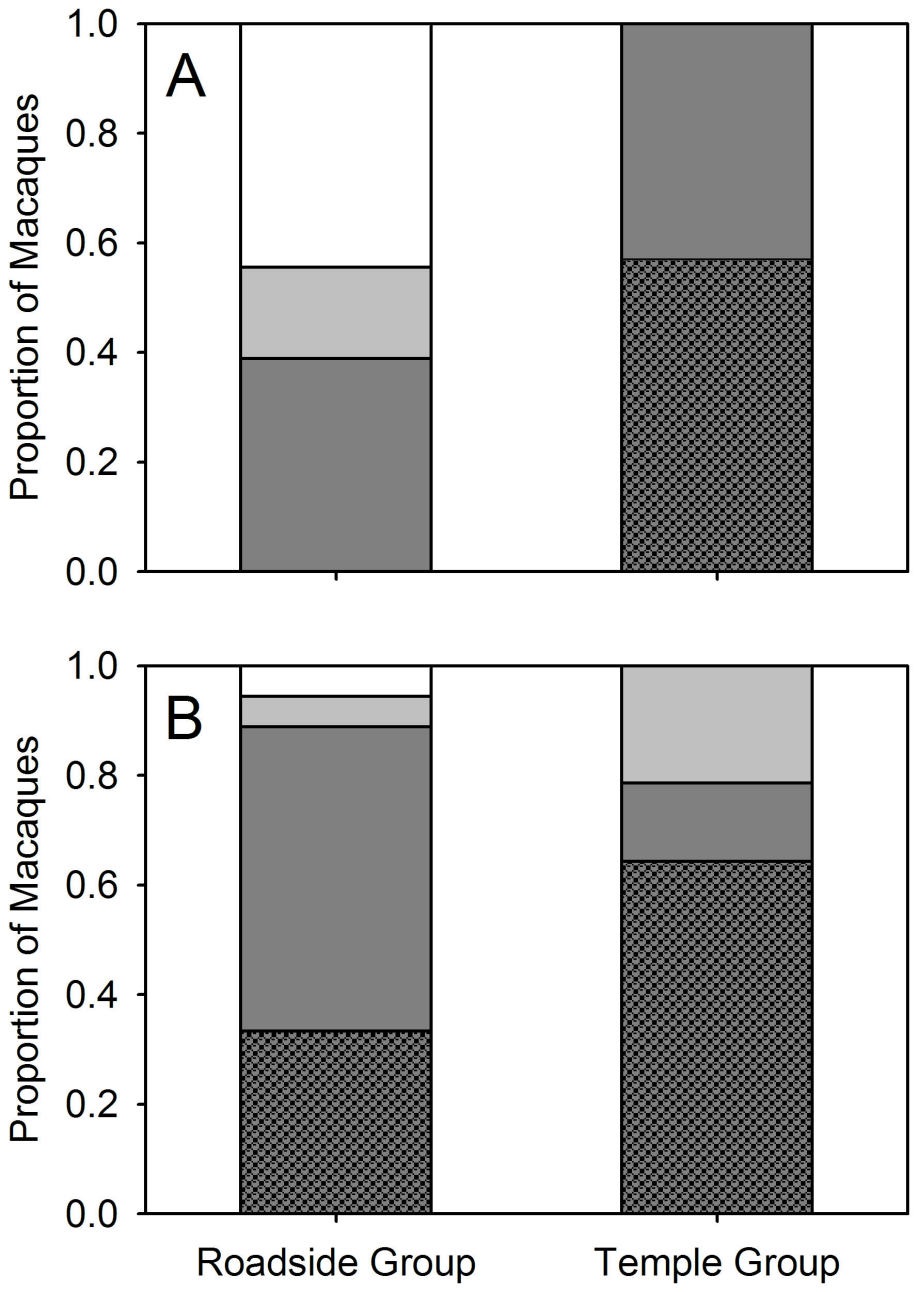
Proportion of Macaques in the Roadside Group (n = 18) and the Temple Group (n = 14) that Explored, Attempted, and Solved the Food Extraction Tasks. (A) Task-1. (B) Task-2. White: ignored grey: explored; dark grey: explored and attempted; patterned dark grey: explored, attempted and solved the task.

In order to examine local differences in food extraction abilities, we used stepwise binary logistic regressions with environment (i.e., the macaque belonging to the roadside or temple group), age-class, and sex as independent categorical predictors and activities including exploration, attempt and success in solving task-1/task-2 as dependent binary variables. Environment significantly predicted problem-solving behavior averaging across the levels of age and sex (Table 1), wherein a significantly greater proportion of the macaques of the temple group (as compared to those of the roadside group) explored, attempted (out of those that explored), and successfully solved (out of those that attempted) task-1 (Figure 1a; see Movies S1 to S4). Among the temple group macaques, only sex had a significant effect on problem-solving success, wherein a significantly greater proportion of females successfully extracted food material from PET bottle, i.e., solved task-1 (Table 1; Fisher’s exact test: males = 1/6, females = 7/8, p = 0.016). Macaques of both groups explored and attempted (out of those that explored) task-2 without any significant differences between them. However, significantly greater proportion of the macaques of the temple group (as compared to those of the roadside group) solved (out of those that attempted) task-2 (Figure 1b; see Movie S5). Among the macaques that extracted food material from the wire mesh box, i.e., solved task-2, the macaques of the temple group solved the task significantly faster as compared to the macaques of the roadside group (Mann-Whitney U test: n_1_ = 6, mean ± se = 186.33 ± 37.14 s, n_2_ = 9, mean ± se = 78.89 ± 6.16, U = 5.000, p = 0.010).

**Table 1:** Results of Binary Logistic Regressions Investigating Predictors of Problem-solving Behavior of the Macaques of the two Groups (n_1_ = 18, n_2_ = 14) When Presented with Task-1 and Task-2.

	**Predictor**	**Task-1**				**Task-2**		
		**χ ^2^**	**df**	**p**		**χ ^2^**	**df**	**p**
Explore (10/16 versus 14/14)								
	Environment	8.296	1	0.004		0.804	1	0.370
	Age-class	2.667	1	0.102		1.031	1	0.310
	Sex	0.711	1	0.399		0.620	1	0.431
Attempt (7/16 versus 14/14)								
	Environment	4.828	1	0.028		1.650	1	0.199
	Age-class	0.098	1	0.754		1.005	1	0.316
	Sex	0.098	1	0.754		0.365	1	0.546
Solve (0/16 versus 8/14)								
	Environment	6.465	1	0.011		5.169	1	0.023
	Age-class	2.032	1	0.154		0.899	1	0.343
	Sex	4.891	1	0.027		2.211	1	0.137

### Between-individual Variation and Within-individual Consistency in Problem-solving Behavior

#### (a): Individual Characteristics and Variation in Problem-solving Success

Table 2 describes problem-solving success/failure (and indicates the solution-techniques) of the macaques of the temple group in the six presentations when presented with task-1 (see Movies S1 to S4). Problem-solving success varied across individuals but remained consistent within each individual across the six presentations of task-1 (Cochran’s Q test: χ ^2^ = 4.999, p = 0.416). In order to examine whether some individual characteristics would have affected problem-solving success of the macaques, we used a stepwise linear regression on the mean problem-solving success (which followed a normal distribution) in the six presentations of task-1 with age-class and sex as independent categorical variables and body mass, dominance status, and sociality as independent continuous variables. Only age-class (t_1,5_ = - 3.126, p = 0.009) and sex (t_1,5_ = 2.405, p = 0.035) significantly predicted problem-solving success of the macaques, wherein adults (mean ± se = 5.500 ± 0.224) solved task-1 significantly more frequently than juveniles (mean ± se = 1.875 ± 0.874) and females (mean ± se = 4.625 ± 0.660) solved task-1 significantly more frequently than males (mean ± se = 1.833 ± 1.667); body mass (t_1,5_ = 0.874, p = 0.402), dominance status (t_1,5_ = - 0.680, p = 0.512), and sociality (t_1,5_ = - 0.449, p = 0.663) did not significantly affect problem-solving success of the macaques.

**Table 2:** Problem-solving Success/Failure of the Macaques of the Temple Group in the Six Presentations When Presented with Task-1.

**Macaque Identity**	**Body Mass (mean ± sd kg)**	**Dominance Status**	**Sociality**	**Presentations**							**Food Extraction Technique**
				**1**	**2**	**3**	**4**	**5**	**6**		
AM1	9.71 ± 0.21	3.29	0.10	✓	✓	✓	✓	✓	✓		Grasping cap with mouth and rotating bottle with both the hands (Movie S1)
AM2	9.22 ± 0.16	2.82	0.26	✗	✓	✓	✓	✓	✓		Puncturing bottle body (Movie S2)
SM1	7.55 ± 0.79	2.30	0.29	✗	✗	✗	✗	✗	✗		Unsuccessful
SM2	5.34 ± 0.06	1.78	0.23	✗	✗	✗	✗	✗	✗		Unsuccessful
JM1	4.12 ± 0.16	1.71	0.18	✗	✗	✗	✗	✗	✗		Unsuccessful
JM2	4.76 ± 0.06	1.84	0.22	✗	✗	✗	✗	✗	✗		Unsuccessful
AF1	6.91 ± 0.17	2.61	0.29	✓	✓	✓	✓	✓	✗		Puncturing bottle neck/body/base
AF2	8.84 ± 0.36	1.65	0.24	✓	✓	✓	✓	✓	✓		Puncturing bottle base (Movie S3)
AF3	6.61 ± 0.10	2.07	0.35	✓	✓	✓	✗	✓	✓		Puncturing bottle shoulder/body/base; grasping cap with mouth and rotating bottle with both the hands
AF4	7.15 ± 0.09	2.65	0.33	✓	✓	✓	✓	✓	✓		Grasping bottle with both the legs and the right hand, and rotating cap with the left hand (Movie S4)
JF1	5.64 ± 0.06	0.56	0.29	✓	✓	✗	✓	✓	·		Puncturing bottle shoulder/base; Grasping bottle with both the legs and rotating cap with both the hands
JF2	4.40 ± 0.07	0.00	0.25	✓	✓	✓	✗	✓	✓		Puncturing bottle neck/shoulder/body
JF3	4.17 ± 0.11	0.99	0.19	✗	✗	✗	✗	✗	✗		Unsuccessful
JF4	3.77 ± 0.09	1.34	0.25	✓	✓	✓	✓	✓	✓		Puncturing bottle neck/shoulder/body

Capital letters indicate individual age-class and sex [A = adult, S = subadult, J = juvenile; M = male, F = female]; ✓ and ✗ indicate success and failure in solving the task; ✓ indicates that the macaque opened bottle cap.

#### (b): Between-individual Variation and Within-individual Consistency in the Solution-technique and Mean Problem-solving Success

In order to examine between-individual variation and within-individual consistency in the solution-technique among the macaques of the temple group, we used likelihood ratio tests which compared ordinal logistic regressions with and without the identity of the focal macaque as a random effect. We assigned numerical values to different solution-techniques, i.e., unsuccessful extraction attempt, ‘0’, puncturing bottle sole, ‘1’, puncturing bottle body, ‘2’, puncturing bottle shoulder, ‘3’, puncturing bottle neck, ‘4’, and opening bottle cap, ‘5’. Solution-technique varied across individuals but remained consistent within each individual across the six presentations of task-1 (Table 2; χ^2^ = 18.415, df = 9, p = 0.031); and with an exception of a few presentations (15 out of 83), the solution-technique (for the macaques that solved task-1), the major solving-technique (for the macaques that failed to solve task-1), and the initial solving-technique remained the same. In order to examine the effect of success and presentations on the problem-solving characteristics, we used mixed-design ANOVAs on each problem-solving characteristic. The macaques that solved task-1 had significantly smaller work-time, which comprised of significantly smaller number of problem-solving bouts, which were significantly shorter in duration, and spent a significantly larger proportion of work-time using the major problem-solving technique, and a significantly smaller proportion of work-time exploring the task apparatus, as compared to the macaques that failed to solve task-1 (Table 3). Neither the order of presentation of task-1, nor the interaction between success and the order of presentation, significantly affect the problem-solving characteristics of the macaques (Table 3).

**Table 3:** Results of Mixed-design ANOVAs on the Problem-solving Characteristics of the Macaques of the Temple Group in the Six Presentations When Presented with Task-1.

	**Predictor**	**f**		**df**	**p**
	Success	9.798		1, 11	0.010
	Presentation	2.272		5, 55	0.060
	Success X Presentation	1.932		5, 55	0.104
	Success	14.331		1, 11	0.003
	Presentation	4.421		5, 55	0.002
	Success X Presentation	1.968		5, 55	0.980
	Success	16.573		1, 27	< 0.001
	Presentation	0.413		5, 135	0.839
	Success X Presentation	0.777		5, 135	0.568
	Success	9.127		1, 11	0.012
	Presentation	1.633		5, 55	0.167
	Success X Presentation	0.352		5, 55	0.878
	Success	15.077		1, 11	0.003
	Presentation	0.579		5, 55	0.716
	Success X Presentation	0.571		5, 55	0.722

Work-time

Number of bouts

Duration of bouts

Proportion of work-time spent on major solution-technique

Proportion of work-time spent on exploration

#### (c): Between-individual Variation and Within-individual Consistency in the Problem-solving Characteristics

**Figure 2:**
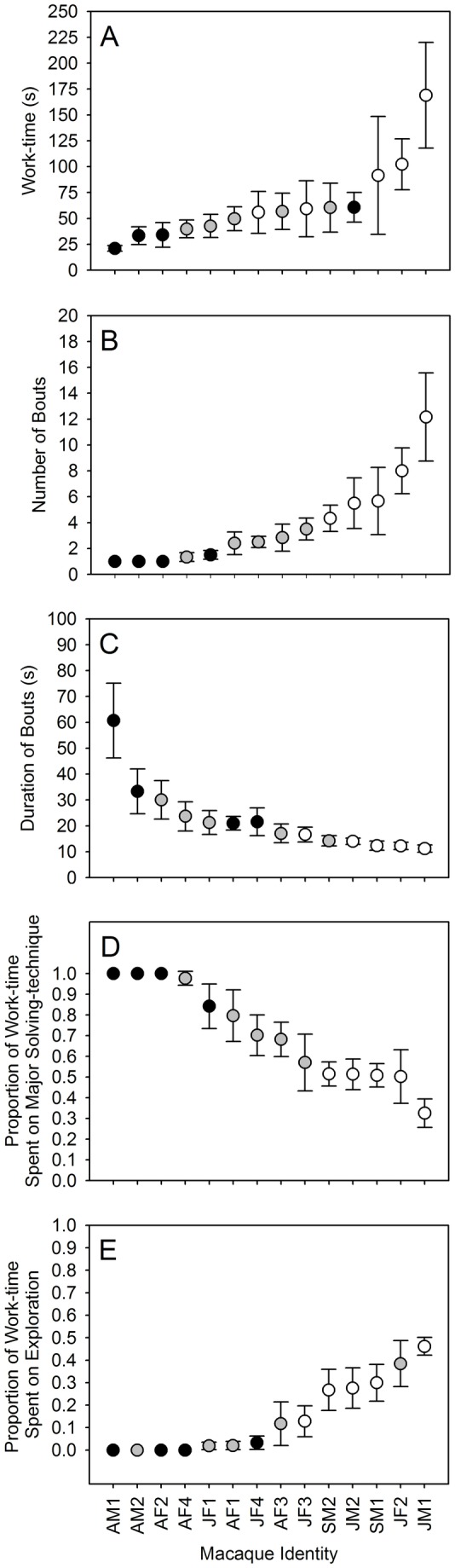
Problem-solving Characteristics of the Temple Group Macaques (n = 14) Across the Six Presentations of Task-1. Mean ± se work-time (A). Number of bouts (B). Duration of bouts (C). Proportion of work-time spent on major problem-solving technique (D). Proportion of work-time spent on exploration (E). Closed black circles: successful; closed grey circles: partially successful; open circles: unsuccessful.

In order to examine between-individual variation and within-individual consistency in the problem-solving characteristics among the macaques of the temple group, we used likelihood ratio tests that compared ordinal logistic regressions with and without the identity of the focal macaque as a random effect. The problem-solving characteristics varied between individuals but remained consistent within each individual across the six presentations of task-1 (Table 4). With regard to each problem-solving characteristic (i.e., work-time, number of bouts, duration of bouts, and proportion of work-time spent using the major problem-solving technique, and proportion of work-time spent exploring the task apparatus), the successful and unsuccessful macaques mostly lie towards the extremes of the range of values, whereas the partially successful macaques lie somewhere in between (Figure 2).

**Table 4:** Results of Ordinal Logistic Regressions Investigating Between-individual Variation and Within-individual Consistency in the Problem-solving Characteristics of the Macaques of the Temple Group in the Six Presentations When Presented with Task-1.

**Problem-solving Characteristic**	**χ ^2^**	**df**	**p**
Work-time	8.592	9	0.037
Number of bouts	16.901	9	0.050
Duration of bouts	18.147	9	0.033
Proportion of work-time spent on major solution-technique	18.317	9	0.032
Proportion of work-time spent on exploration	8.109	9	0.523

### Behavioral Patterns Associated with Higher Efficiency in Food Extraction

Food extraction behavior of the two macaques of the temple group, AM1 and AF4, regarding the manipulated versions of task-1, is summarized in Table 5. Among the 9 macaques of the temple group that solved task-1 previously, only AM1 and AF4 solved task-3 (their solution-techniques were the same as the one while solving task-1, i.e., rotating the cap), whereas the others failed to solve task-3 even after spending significantly more time than that they did solving task-1 previously (Wilcoxon signed rank test: n = 7, T = - 2.197, p = 0.028).

**Table 5:** Problem-solving Techniques of the two Macaques of the Temple Group, AM1 and AF4, When Presented with the Modified Versions of Task-1.

**Task**		**Solution-technique/Solving-technique**		
		**AM1**		**AF4**
Task-1: non-manipulated (experiment 2)		opened cap by first loosening it with mouth and then rotating it with hand		tore off cap-seal with mouth and opened cap by rotating it with hand
Task-1a: without cap-seal		–		opened cap by rotating it with hand
Task-1b: smaller cap-seal		–		opened cap by rotating it with hand
Task-1c: immovable cap and cap-seal		unable to rotate or manipulate cap with mouth, abandoned the task		unable to tear off cap-seal, abandoned the task
Task-1d: non-functional cap and cap-seal		rotated cap with mouth and afterwards with hand, and then tore off both cap and cap-seal		after rotating cap with hand for some time, abandoned the task

## Discussion

We observed considerable local differences in food extraction abilities, and between-individual variation and within-individual consistency in food extraction techniques among free-ranging bonnet macaques. When presented with novel food extraction tasks, the macaques that were more frequently exposed to novel physical objects in their surroundings (i.e., the macaques of the temple group), extracted food material from PET bottles and also solved another food extraction task (i.e., extracting an orange from a wire mesh box), more often than those living under more natural conditions (i.e., the macaques of the roadside group). Adults solved the task more frequently than juveniles, and females more frequently than males. The solution-technique and problem-solving characteristics varied between individuals but remained consistent within each individual across the successive presentations of PET bottles. The macaques that solved the tasks showed lesser within-individual variation in their food extraction behavior as compared to those that failed to solve the tasks. A few macaques appropriately modified their problem-solving behavior in accordance with the task requirements and solved the modified versions of the tasks without trial-and-error learning. These observations are ecologically relevant as the observed flexibility in food extraction techniques is likely to affect the species’ local adaptability and resilience to environmental changes.

In urban environments, anthropogenic discard potentially represents a substantial portion of macaques’ dietary intake, but typically macaques have a restricted access to these resources because of some packaging (personal observation). Macaques, when confronted with embedded food resources more often than those that are relatively simple to process, are likely to develop appropriate foraging (here, food extraction) abilities to meet their daily dietary requirements. Consistent with this, the macaques of the temple group explored, attempted and solved task-1 more frequently as compared to the macaques of the roadside group. If the observed differences between the macaques of the two groups regarding task-1 reflect the differences in their responses towards novelty, we would expect them to show similar differences also regarding task-2 (presumably, a task which was a novel one for the macaques of both groups). Although the macaques of the temple group solved task-2 more often than the macaques of the roadside group, the macaques of the two groups exhibited no variation or difference in exploring and attempting task-2. Perhaps the macaques of the temple group developed food extraction abilities which facilitated their use of novel, embedded food resources, enhancing their survival under more variable environmental conditions. Observations on several animal species, for example, feral pigeons, *Columba livia*, mourning dove, *Zenaida macroura* [32], wild vervet monkeys, *Chlorocebus aethiops* [33], bearded capuchin monkeys, *Cebus libidinosus* [34] (now known as *Sapajus libidinous* [35,36]), tufted capuchin monkeys, *Cebus apella* [37] (now known as *Sapajus apella* [35,36]), and chimpanzees [38], suggest that individuals with more frequent exposure to novel physical objects in their surroundings may generally show reduced neophobic responses and hence, partake in and solve novel food extraction tasks more often than those living under more natural conditions. Accordingly, the observed patterns of local differences in problem-solving behavior between the macaques of two groups are most likely to be influenced by the differences in the physical structure of their surroundings.

Within the macaques of the temple group, adults extracted food material from PET bottles, i.e., solved task-1, more often than juveniles, and females more often than males. We explain these age-class and sex related differences the way Reader and Laland [17] explain the previously reported foraging specializations in non-human primates. The development of food extraction abilities require pre-acquired knowledge and skills; thus, experienced adults may be more likely than less experienced juveniles to successfully exploit food resources which require specialized extraction techniques. As the socio-ecological theory suggests that reproductive success of females (and not that of males) is directly linked with access to food resources [39], females may be more likely than males to opt for alternative foraging strategies, which increases the magnitude and predictability of returns from foraging. Alternatively, as body mass is often correlated with competitive abilities (see, for example, observations on stone tool use by bearded capuchin monkeys, [22]) and dominance status determines priority of access to resources among macaques [40], it follows that the typically lightweight and low-ranking females [41] may be more likely than the males to opt for embedded food resources; however, body mass or dominance status did not affect problem-solving success of the macaques of the temple group and therefore this explanation remains propositional.

The macaques of the temple group that solved task-1 in the first presentation continually solved it over the successive presentations, whereas the macaques that failed to solve task-1 in the first presentation mostly failed to solve it in the successive presentations. Only a few macaques (AF3 and JF1) developed the ability to open bottle cap on the fifth presentation, showing that they learned something about the device features and how they work during the presentations. The solution-technique (or the major solving-technique for those macaques that failed to solve task-1) and the problem-solving characteristics remained consistent within and between the macaques of the temple group across the six presentations of task-1. As mentioned earlier, this kind of correlated set of individual behavioral traits, which remain consistent over time and situations, confers differential fitness consequences under divergent environmental conditions [26-28]. Between-individual variation and within-individual consistency in food extraction abilities is an aspect of behavioral flexibility that seems to be correlated with the individual level of innovative propensity (as defined by Reader and Laland [42]), suggesting that novel problem-solving ability can be considered as a fundamental and presumably stable component of individuals’ responses to environmental heterogeneity. Enhanced problem-solving ability may not only affect the way individuals respond to their social and physical environments, but also the way they respond to variations in the food resource availability.

Among the macaques of the temple group, the individuals that solved task-1 had a smaller work-time, which comprised of a smaller number of problem-solving bouts, which were shorter in duration, and spent a larger proportion of work-time using the major problem-solving technique, and a smaller proportion of work-time exploring the task apparatus, as compared to the individuals that failed to solve task-1. Overall, the macaques that solved task-1 exhibited lesser within-individual variation in their food extraction behavior as compared to those that continually failed to solve task-1. Perhaps the macaques of the temple group, during their regular encounters with the PET bottles, learned to extract food material from them through trial-and-error, and later improved upon their extraction techniques or adopted more effective techniques. In this case, the observed between-individual variations in food extraction abilities among the macaques of the temple group are likely to be a manifestation of the underlying social and /or asocial learning processes. However, both the design and the scope of our study do not allow us to comment on any of these processes.

When presented with the manipulated versions of task-1, the two macaques of the temple group, AM1 and AF4, which solved task-1 earlier by opening bottle cap, solved them without trial-and-error learning. AM1, which typically loosened the cap in task-1 with mouth followed by rotation with hand, abandoned task-1c after failing to loosen the immovable cap with mouth, and tore off both the cap and cap-seal in task-1d after rotating them for some time with mouth followed by hand. AF4, which typically opened the cap in task-1 by first tearing off the cap-seal with mouth and then rotating the cap with hand, directly opened the cap in task-1a and task-1b with hand without even looking for cap-seal, and abandoned task-1c and task-1d after failing to tear off the cap-seal in task-1c and after rotating the cap in task-1d for some time with hand, respectively. These observations show flexibility and consistency in problem-solving. As shown from these observations, both the macaques excluded the action patterns (which they used while solving task-1 earlier) that could have proved counterproductive while solving the modified versions of task-1. Moreover, AM1 and AF4 were the only two macaques of the temple group that solved task-3 – a task which was functionally similar to task-1. Perhaps the macaques discovered the food material content during their encounters with the uncapped PET bottles found in discard, and subsequently, relatively more exploratory individuals developed the effective solution-technique(s). 

The motor actions during novel problem solving might reflect an individual’s natural exploratory behavior and routine (which may vary across species). For example, capuchin monkeys regularly extract food material from embedded resources [22,43,44], and they show exploratory routine, such as tapping and percussive behavior, for this activity [45]; thus, capuchins might approach the tasks which we used in our study very differently. However, in the present study, the macaques inhabiting more natural habitat did not show any behavior which even remotely resembled the behavior of the macaques with more frequent exposure to novel physical objects in their surroundings (e.g., the motor actions involved in opening bottle cap – tearing off the cap-seal with mouth and/or rotating the cap with hand or mouth). This suggests modified exploratory behaviour in the macaques that inhabited the more human influenced environment as compared to their counterparts that inhabited the more natural habitat.  One can expect the emergence and evolution of individual specializations in foraging behavior (which, in the context of the present study, concerns exploitation of anthropogenic food resources) owing to a diverse array of physiological, behavioral, and ecological mechanisms [4-6].. We propose further investigations on foraging specializations in non-human primates by observing behavior of individuals (a) across a wide range of natural /spontaneous foraging activities, and (b) across experimental tasks that require dexterity of different kinds, both under different free-ranging conditions.

## Supporting Information

Figure S1
**Task-1, an Unsealed PET Bottle Containing c.a. 50 ml Sweet Milk.**
(TIF)

Figure S2
**Task-2, a Wire Mesh Box Containing an Orange.**
(TIF)

Figure S3
**Manipulated versions of task 1.** Task-1a, an unsealed PET bottle without cap-seal (A). Task-1b, an unsealed PET bottle with a smaller cap seal (B). Task-1d, a PET bottle with immovable cap and cap-seal (C). Task 1-d, a PET bottle with non-functional cap and cap-seal (D). (TIF)

Figure S4
**Task-3, a polycarbonate water bottle containing c.a. 50 ml sweet milk.**
(TIF)

Movie S1
**This Footage Illustrates an Adult Male Bonnet Macaque (AM1, Temple Group) Solving Task-1.** The macaque grasps cap with mouth and opens bottle by rotating it with both the hands. (MP4)

Movie S2
**This Footage Illustrates an Adult Male Bonnet Macaque (AM2, Temple Group) Solving Task-1.** The macaque punctures bottle-body, spills the liquid by rolling bottle with both the hands and licks the spill. (MP4)

Movie S3
**This Footage Illustrates an Adult Female Bonnet Macaque (AF2, Temple Group) Solving Task-1.** The macaque punctures bottle-base and drinks the liquid by sucking it through the puncture. (MP4)

Movie S4
**This Footage Illustrates an Adult Female Bonnet Macaque (AF4, Temple Group) Solving Task-1.** The macaque first attempts to open cap by rotating it with the left hand, failing to do so, it tears off bottle cap seal with mouth, grasps bottle with both the legs and the right hand and opens cap by rotating it with the left hand. (MP4)

Movie S5
**This Footage Illustrates a Juvenile Female Bonnet Macaque (JF4, Temple Group) Solving Task-2.** The macaque opens lid of the cage using the left leg and the right hand and takes out orange with the left hand. (MP4)
