# Correction: Flexibility in Food Extraction Techniques in Urban Free-Ranging Bonnet Macaques, Macaca radiata

**DOI:** 10.1371/journal.pone.0091480

**Published:** 2014-03-12

**Authors:** The PLOS ONE Staff

The publisher apologizes for the following errors, which were introduced during the production process.

Supplementary figure titles and legends, as well as movie titles and legends, do not appear in the PDF version of the article, but do appear in the online version. Please view the figure and movie legends below.

In Table 3, the titles are shifted to footnotes. Please view the correct Table 3 here:

**Table 3:** Results of Mixed-design ANOVAs on the Problem-solving Characteristics of the Macaques of the Temple Group in the Six Presentations When Presented with Task-1.

Predictor	f	df	p
*Work-time*			
Success	9.798	1, 11	0.010
Presentation	2.272	5, 55	0.060
Success X Presentation	1.932	5, 55	0.104
*Number of bouts*			
Success	14.331	1, 11	0.003
Presentation	4.421	5, 55	0.002
Success X Presentation	1.968	5, 55	0.980
*Duration of bouts*			
Success	16.573	1, 27	< 0.001
Presentation	0.413	5, 135	0.839
Success X Presentation	0.777	5, 135	0.568
*Proportion of work-time spent on major solution-technique*			
Success	9.127	1, 11	0.012
Presentation	1.633	5, 55	0.167
Success X Presentation	0.352	5, 55	0.878
*Proportion of work-time spent on exploration*			
Success	15.077	1, 11	0.003
Presentation	0.579	5, 55	0.716
Success X Presentation	0.571	5, 55	0.722

## Supporting Information

Figure S1 **Task-1, an Unsealed PET Bottle Containing c.a. 50 ml Sweet Milk.**
(TIF)

Figure S2 **Task-2, a Wire Mesh Box Containing an Orange.**
(TIF)

Figure S3 **Manipulated versions of task 1.** Task-1a, an unsealed PET bottle without cap-seal (A). Task-1b, an unsealed PET bottle with a smaller cap-seal (B). Task-1d, a PET bottle with immovable cap and cap-seal (C). Task 1-d, a PET bottle with non-functional cap and cap-seal (D).
(TIF)

Figure S4 **Task-3, a polycarbonate water bottle containing c.a. 50 ml sweet milk.**
(TIF)

Movie S1 **This Footage Illustrates an Adult Male Bonnet Macaque (AM1, Temple Group) Solving Task-1.** The macaque grasps cap with mouth and opens bottle by rotating it with both the hands.
(MP4)

Movie S2 **This Footage Illustrates an Adult Male Bonnet Macaque (AM2, Temple Group) Solving Task-1.** The macaque punctures bottle-body, spills the liquid by rolling bottle with both the hands and licks the spill.
(MP4)

Movie S3 **This Footage Illustrates an Adult Female Bonnet Macaque (AF2, Temple Group) Solving Task-1.** The macaque punctures bottle-base and drinks the liquid by sucking it through the puncture.
(MP4)

Movie S4 **This Footage Illustrates an Adult Female Bonnet Macaque (AF4, Temple Group) Solving Task-1.** The macaque first attempts to open cap by rotating it with the left hand, failing to do so, it tears off bottle cap-seal with mouth, grasps bottle with both the legs and the right hand and opens cap by rotating it with the left hand.
(MP4)

Movie S5 **This Footage Illustrates a Juvenile Female Bonnet Macaque (JF4, Temple Group) Solving Task-2.** The macaque opens lid of the cage using the left leg and the right hand and takes out orange with the left hand.
(MP4)
