# Supplementary figures and images for: Correction: Flexibility in Food Extraction Techniques in Urban Free-Ranging Bonnet Macaques, Macaca radiata
Source: PLoS One. 2014 Mar 12;9(3):e91480. doi: 10.1371/journal.pone.0091480 (PMC3951384; doi:10.1371/journal.pone.0091480)

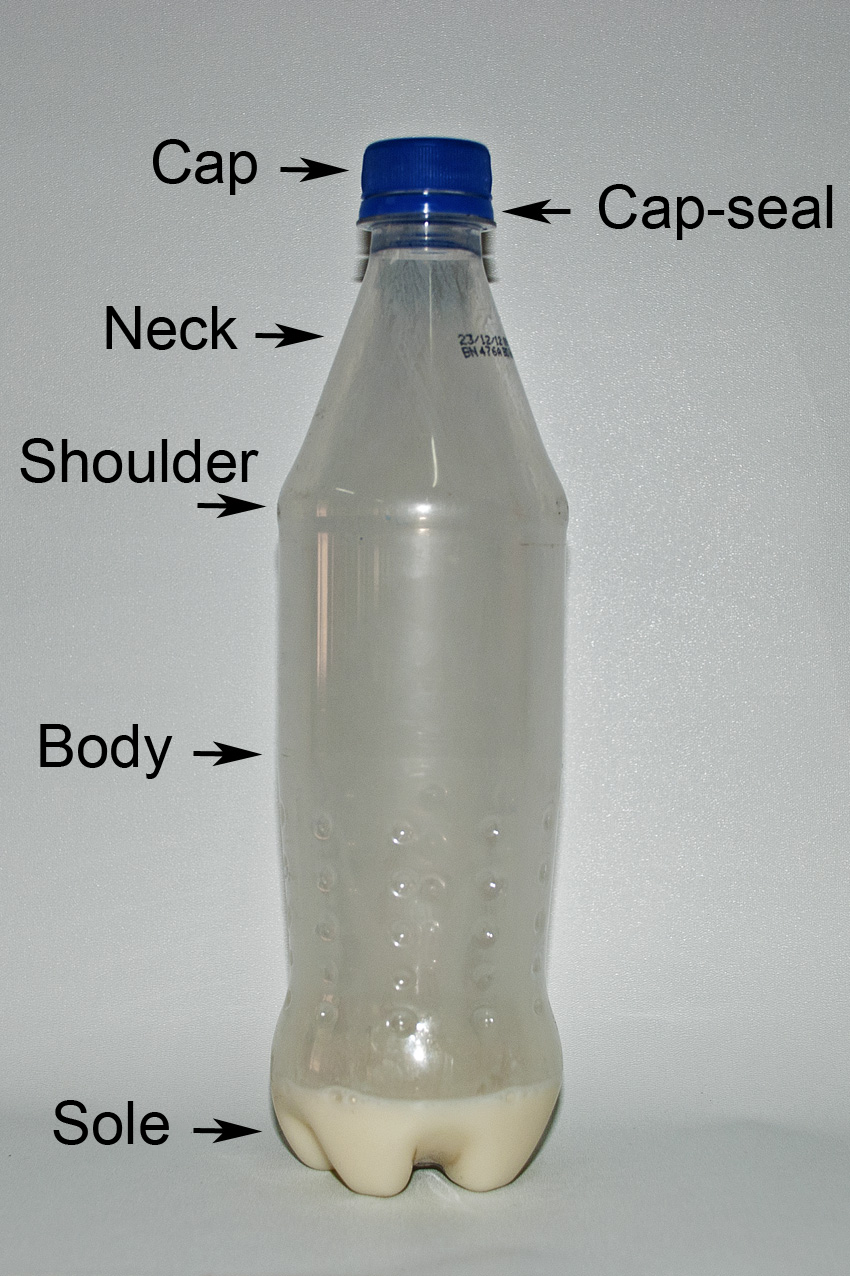

Supplement: Figure S1 — Task-1, an Unsealed PET Bottle Containing c.a. 50 ml Sweet Milk. (TIF) [file pone.0091480.s001.tif]

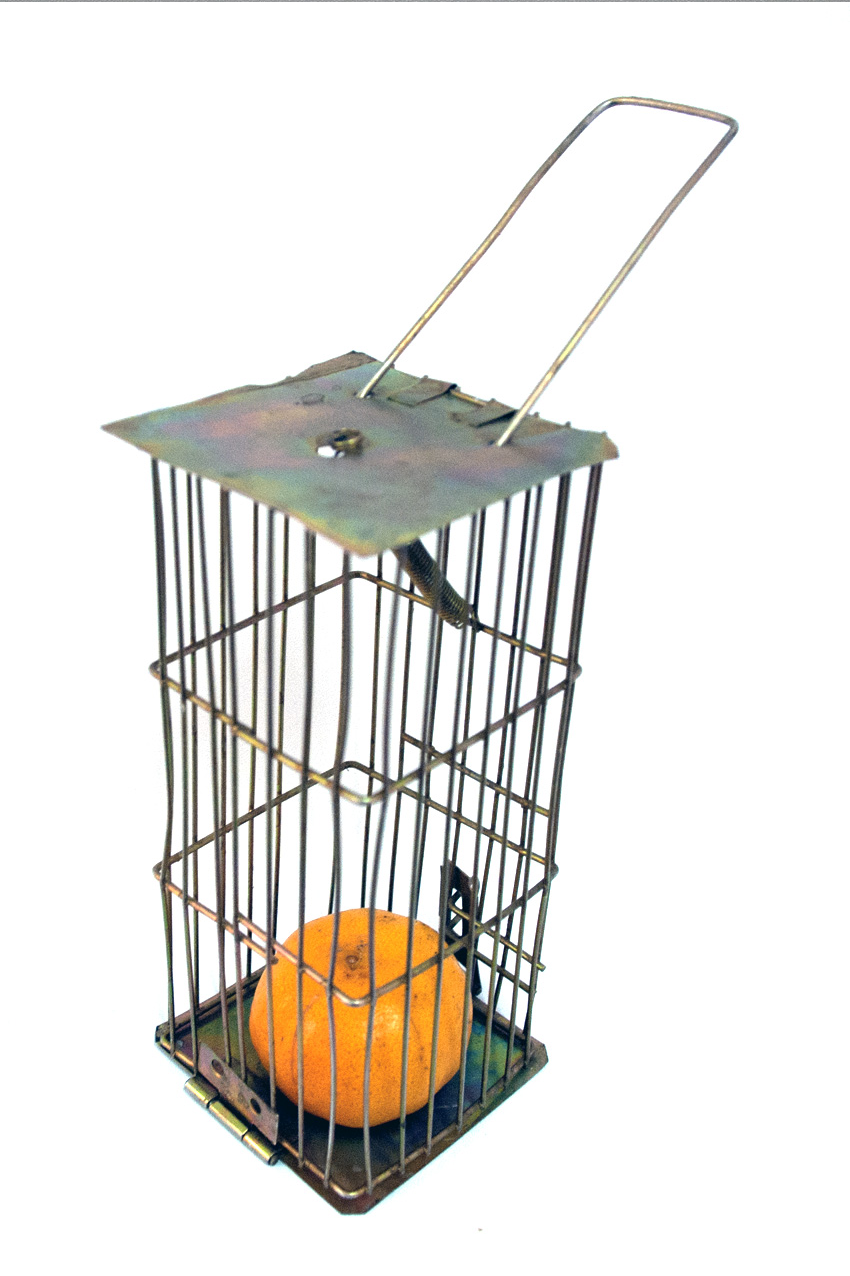

Supplement: Figure S2 — Task-2, a Wire Mesh Box Containing an Orange. (TIF) [file pone.0091480.s002.tif]

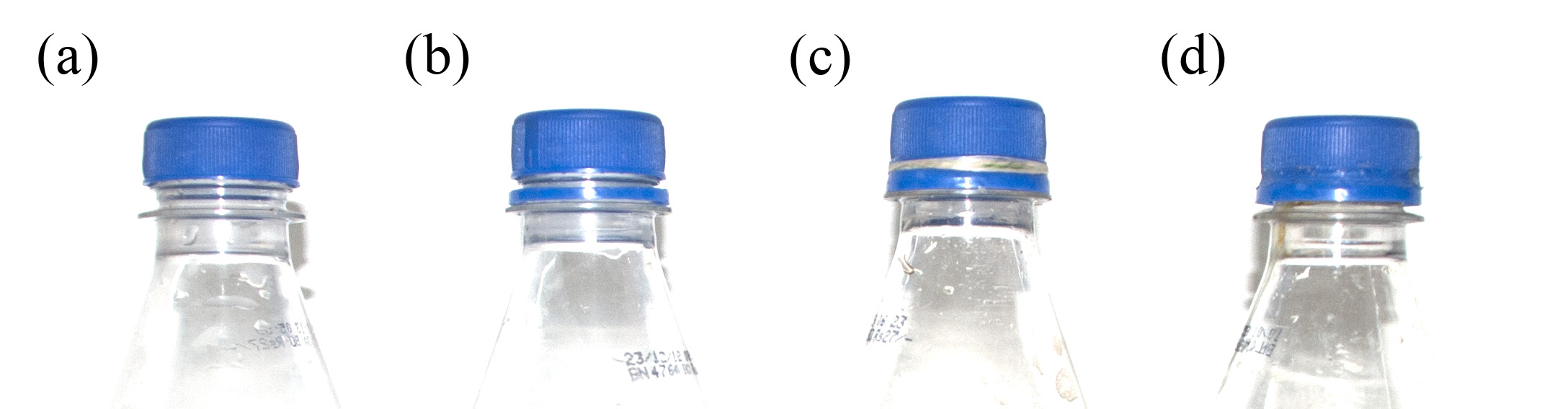

Supplement: Figure S3 — Manipulated versions of task 1. Task-1a, an unsealed PET bottle without cap-seal (A). Task-1b, an unsealed PET bottle with a smaller cap-seal (B). Task-1d, a PET bottle with immovable cap and cap-seal (C). Task 1-d, a PET bottle with non-functional cap and cap-seal (D). (TIF) [file pone.0091480.s003.tif]

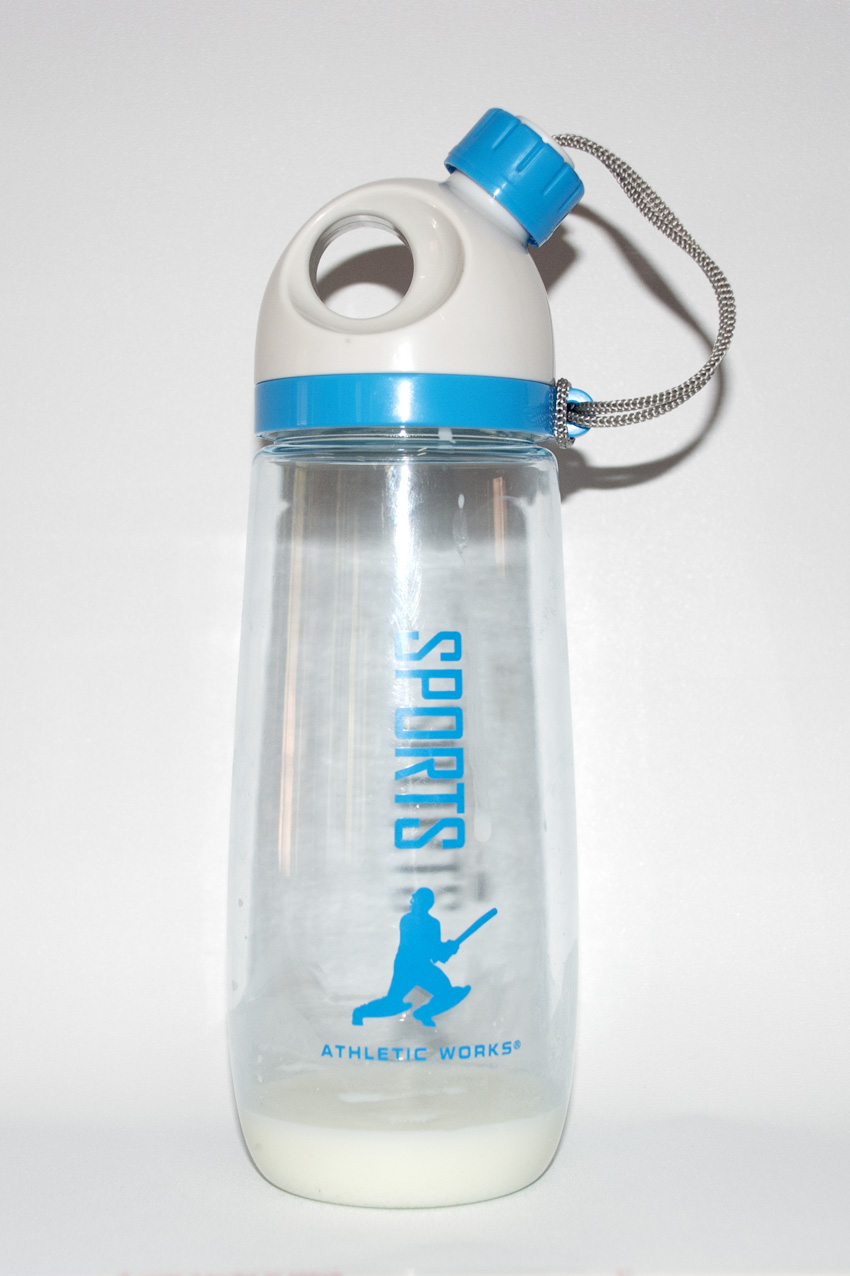

Supplement: Figure S4 — Task-3, a polycarbonate water bottle containing c.a. 50 ml sweet milk. (TIF) [file pone.0091480.s004.tif]
